# Hematological Biomarkers in Predicting Progression and Complications of Chronic Kidney Disease Among Adult Patients

**DOI:** 10.1155/ah/8487716

**Published:** 2025-12-04

**Authors:** Collince Odiwuor Ogolla, Lucy W. Karani, Stanslaus Musyoki, Phidelis Maruti

**Affiliations:** ^1^ Department of Applied Health Science, School of Health Science, Kisii University, P.O. Box 408-40200, Kisii, Kenya, kisiiuniversity.ac.ke; ^2^ Department of Medical Laboratory Science, School of Health Science, South Eastern Kenya University, P.O. Box 170-90200, Kitui, Kenya, seku.ac.ke; ^3^ Department of Medical Laboratory Science, School of Health Science, Kisii University, P.O. Box 408-40200, Kisii, Kenya, kisiiuniversity.ac.ke

**Keywords:** anemia, chronic kidney disease (CKD), hematological biomarkers, hemoglobin

## Abstract

**Background:**

CKD is a progressive disorder that is commonly associated with hematological abnormalities.

**Objective:**

The objective of the study was to evaluate the role of hematological biomarkers in predicting CKD progression and related complications in adult patients.

**Methods:**

The retrospective cross‐sectional study evaluated hematological parameters such as hemoglobin, RBC, WBC, platelet, and NLR; comorbidities; and CKD stages were recorded for analysis. Statistical analysis methods used included ANOVA, Chi‐square, multiple logistic regression, Pearson correlation analyses, and ROC curve analysis. The study was conducted under strict adherence to the principles and guidelines of the Helsinki Declaration (2013).

**Results:**

The mean age was 56.2 ± 14.8 years, with males being 56.7%. About 70.8% of the patients were in CKD Stages 3–5. Anemia was observed in 74.2% of the patients, whose prevalence increased alongside the increase in severity of CKD (*p* < 0.001). There was a significant decrease in hemoglobin, RBC, and platelet counts with advancing CKD stages, whereas WBC and NLR increased (*p* < 0.001). Hemoglobin (OR: 0.72; *p* < 0.001), NLR (OR: 1.43; *p* = 0.006), and platelet count (OR: 0.98; *p* = 0.021) were independent predictors of progression to CKD Stage 5. ROC analysis yielded good results for hemoglobin (AUC: 0.81) and NLR (AUC: 0.76) in predicting CKD Stage 5. Hemoglobin and platelet‐count levels were significantly correlated with eGFR (*r* = −0.70 and *r* = 0.58, respectively).

**Conclusion:**

The performance of hematological biomarkers, mainly hemoglobin and NLR, emerges as reliable predictor of CKD progression and complications. Their assessment as part of the CKD workup may then enhance risk stratification and early intervention.

**Summary**

• The current study brings attention to some routine hematological biomarkers for the prediction of chronic kidney disease (CKD) progression and complications‐with hemoglobin (Hb) and neutrophil‐to‐lymphocyte ratio (NLR) taking the lead.
• It proves, with a sub‐Saharan African population as example, that such cheap blood tests could be included in the risk stratification and early intervention strategies for CKD.

## 1. Introduction

CKD is a worldwide public health problem affecting millions at present [1]. It is estimated by the World Health Organization that around 13% of the global population suffers from CKD and that its prevalence continues to rise due to hypertension, diabetes, and an aging populace [2]. CKD usually involves a progressive and irreversible loss of renal function, which if left untreated may finally lead to end‐stage kidney disease (ESKD) with a need for either dialysis or kidney transplantation. An early detection of such patients identified as being at high risk of progression is essential to timely intervention and better patient outcomes [3].

Some changes in blood are observed that include reduction in red blood cells (RBCs), platelet counts, or typical alterations in white blood cell (WBC) counts [1]. Anemia sets in for the majority of CKD patients, especially those that have aggravated CKD, while it is also associated with worse cardiovascular conditions [4]. Markers of inflammation such as NLR have been characterized as factors that impact CKD progression and some associated complications such as left ventricular hypertrophy (LVH) and fluid overload [5]. NLR was included as it provides a simple, inexpensive measure of systemic inflammation and has been increasingly associated with cardiovascular morbidity and CKD progression. These markers could be showing processes that worsen CKD morbidity‐anemia, vascular insult, and generalized inflammation.

Moreover, hematological abnormalities in CKD patients not only reflect the underlying renal impairment but also lead directly to increased morbidity and mortality [6]. For instance, anemia worsens tissue hypoxia, ramping up cardiovascular threshing—the number one cause of death in CKD worldwide [7]. In the same trajectory, thrombocytopenia accompanied by platelet dysfunction increases hemorrhagic tendencies, thus complicating clinical management, whereas leukocytosis and raised inflammatory markers such as NLR could be indicative of systemic inflammation and oxidative stress; these two being pivotal factors in progressing CKD and cardiovascular disease [8, 9].

Despite the well‐known pathophysiological basis, translating this knowledge into readily available, cheap predictive tools is still lacking, especially in the sub‐Saharan African healthcare environment where there is inadequate availability of resources for special diagnostic work‐up [10]. Due to economic constraints and limited access to specialized nephrology services, the interest is placed on simple yet dependable biomarker units that can be measured in all primary and secondary care facilities to identify patients at risk of rapid CKD progression and adverse outcomes [11].

Furthermore, the heterogeneous nature of CKD etiology and progression patterns in African populations, which is further influenced by genetic predisposition factors, infectious and noninfectious agents, and late presentation, calls for investigations specific to this region to validate hematological biomarkers’ predictive value [12, 13]. Modification of risk assessment instruments to match this particular group is essential to boost detection timing and treatment efficiency and minimize CKD complication severity in these populations [14].

The research aimed to fill existing knowledge vacuums while simultaneously establishing new standards for medical treatments applicable to under‐resourced settings. The study results have the potential to create new screening processes and establish hematological marker monitoring as a standard practice for CKD management which would improve patient care strategies and health outcomes in various hospitals and comparable regional environments. Despite considerable evidence pointing to some hematological parameters affected by CKD, very few studies have gone ahead to assess their role as useful predictive factors in low‐resource settings. The majority of published literature appears to make reference to high‐income countries where such elaborate diagnostic procedures can be found. In addition, given the meager resources in sub‐Saharan Africa, where CKD is said to be on the rise, we thus require cheap biomarkers for the early detection of disease progression from their perspective. Finding a link between these markers and CKD stages will help in identifying crude markers as a value addition to enhance CKD management, especially in resource‐limited settings.

## 2. Methods

### 2.1. Ethical Considerations

Data confidentiality was achieved through the deidentification and anonymization of data at the time of its extraction. Study investigators only had access to identifiable patient information. All ethical procedures were in line with the Declaration of Helsinki (2013) and WHO guidelines on research involving human subjects.

### 2.2. Study Design

This was a retrospective cross‐sectional study conducted objectively to evaluate the role of hematological biomarkers in predicting progression and complications in adult patients diagnosed with CKD.

### 2.3. Study Population and Sampling

One hundred and twenty adult patients (aged ≥ 18 years) diagnosed with CKD and having full hematological and clinical records were included in the study. Data extraction was carried out from archived patient records. A census sampling method was used that included all eligible patients within the 1‐year review period who met inclusion criteria.

### 2.4. Inclusion and Exclusion Criteria

Inclusion criteria included adult patients (≥ 18 years) with a confirmed diagnosis of CKD (Stages 1–5) and complete medical and hematological records within the study period. Exclusion criteria included patients with incomplete records, pediatric cases (< 18 years), or those with known hematological malignancies or acute infections unrelated to CKD at the time of sampling.

## 3. Data Collection and Variables

Data were gathered from patient files, electronic health records (EHRs), and laboratory information systems. Variables accounted for were as follows: demographic data: age and gender; clinical variables: CKD stage, presence of hypertension, diabetes, or cardiovascular complications. Hematological parameters included Hb, RBC count, WBC count, platelet count, and NLR. CKD staging was done based on the Kidney Disease: Improving Global Outcomes (KDIGO) 2021 classification using estimated glomerular filtration rate (eGFR) [15]. CKD staging criteria: CKDs were staged according to the values of eGFR calculated by Chronic Kidney Disease Epidemiology Collaboration (CKD‐EPI) formula 2021 [16]. Patients were classified according to the KDIGO guidelines in the following manner: Stage 1: eGFR ≥ 90 mL/min/1.73 m^2^ with evidence of kidney damage (e.g., albuminuria), Stage 2: eGFR 60–89 mL/min/1.73 m^2^, Stage 3: eGFR 30–59 mL/min/1.73 m^2^, Stage 4: eGFR 15–29 mL/min/1.73 m^2^, and Stage 5: eGFR < 15 mL/min/1.73 m^2^ or dialysis‐dependent. eGFR values were gathered from laboratory reports included within the patients’ medical record.

## 4. Statistical Analysis

All analyses were performed using IBM SPSS Statistics Version 26.0 (IBM Corp., Armonk, NY, USA). Descriptive statistics were calculated with means and standard deviation (SD) for continuous variables and frequencies and percentages for categorical variables. To compare hematological biomarkers in different CKD stages, one‐way analysis of variance (ANOVA) was applied. Chi‐square tests were conducted for categorical comparisons such as anemia prevalence across CKD stages. To determine the predictors of advanced CKD (Stage 5), multiple logistic regression was developed. For independent variables, Hb, NLR, and platelet count were considered. Adjusted odds ratios with 95% confidence intervals (CIs) were offered. All logistic regression models were adjusted for potential confounders including age, sex, hypertension, and diabetes mellitus. The receiver operating characteristic (ROC) curves determined the discriminative ability of the selected hematological biomarkers for CKD progression, with the area under the curve (AUC) corresponding to Hb, NLR, and platelets. The Pearson correlation coefficients analyzed the relationship between biomarkers and kidney function (eGFR), with significance set at *p* < 0.005.

## 5. Results

### 5.1. Demographic and Clinical Characteristics

The study enrolled 120 adult CKD patients. The average age of the participants was 56.2 ± 14.8 years, with a male‐predominant gender ratio of 1.3:1 (68 male [56.7%] and 52 female [43.3%]). Most patients (85 or 70.8%) were recorded as being in CKD Stages 3–5. Comorbid conditions were quite frequent, with 72 patients (60%) having hypertension, 39 (32.5%) having diabetes mellitus, and 24 (20%) being affected by both. The remaining 27 (22.5%) had no documentation of comorbidity as illustrated in Table 1.

**Table 1:** Demographic and clinical characteristics of the study population (*N* = 120).

Variable	Value
Age (mean ± SD)	56.2 ± 14.8 years
Sex	
Male	68 (56.7%)
Female	52 (43.3%)
CKD Stage	
Stage 1	18 (15.0%)
Stage 2	17 (14.2%)
Stage 3	28 (23.3%)
Stage 4	26 (21.7%)
Stage 5	31 (25.8%)
Comorbidities	
Hypertension	72 (60.0%)
Diabetes mellitus	39 (32.5%)
Both hypertension and diabetes	24 (20.0%)
None	27 (22.5%)

### 5.2. Hematological Parameters by CKD Stage

**Figure 1:**
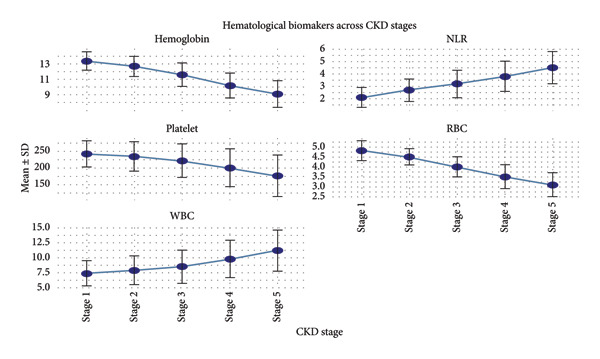
Hematological biomarkers across CKD stages.

The Hb levels demonstrated a significant decrease with increasing CKD stage. The mean Hb level in Stage 1 was 13.4 ± 1.2 g/dL and fell to 9.1 ± 1.7 g/dL in Stage 5 (*p* < 0.001). RBC counts also progressively dropped from 4.8 ± 0.5 × 10^6^/μL in the early stages to 3.1 ± 0.6 × 10^6^/μL in later stages. WBC counts were higher in the patients presented with infection or inflammation (e.g., urinary tract infection, sepsis, or sepsis‐complications) with mean WBC levels at 11.2 ± 3.4 × 10^3^/μL versus 7.8 ± 2.6 × 10^3^/μL WBC for stable patients (*p* = 0.004). Meanwhile, platelet counts differed, with thrombocytopenia (platelets < 150 × 10^3^/μL) being higher in Stage 5 CKD at 26.7% compared with 5.6% in Stage 1 as illustrated in Table 2 and Figure 1.

**Table 2:** Hematological parameters across CKD stages.

CKD stage	*n*	Hemoglobin (g/dL)	RBC (× 10^6^/μL)	WBC (× 10^3^/μL)	Platelets (× 10^3^/μL)	Anemia (%)
Stage 1	18	13.4 ± 1.2	4.8 ± 0.5	7.4 ± 2.1	242 ± 39	33.3
Stage 2	17	12.7 ± 1.3	4.5 ± 0.4	7.9 ± 2.4	234 ± 44	52.9
Stage 3	28	11.6 ± 1.5	4.0 ± 0.5	8.5 ± 2.8	221 ± 51	67.9
Stage 4	26	10.2 ± 1.6	3.5 ± 0.6	9.8 ± 3.1	199 ± 57	84.6
Stage 5	31	9.1 ± 1.7	3.1 ± 0.6	11.2 ± 3.4	176 ± 63	92.9
*p* value	—	< 0.001	< 0.001	0.004	0.011	< 0.001

Figure 1 shows the mean (±SD) values of Hb, platelets, RBC, NLR, and WBC across CKD Stages 1–5. Hb, platelet count, and RBC progressively decline, while NLR and WBC increase with advancing CKD severity.

**Figure 2:**
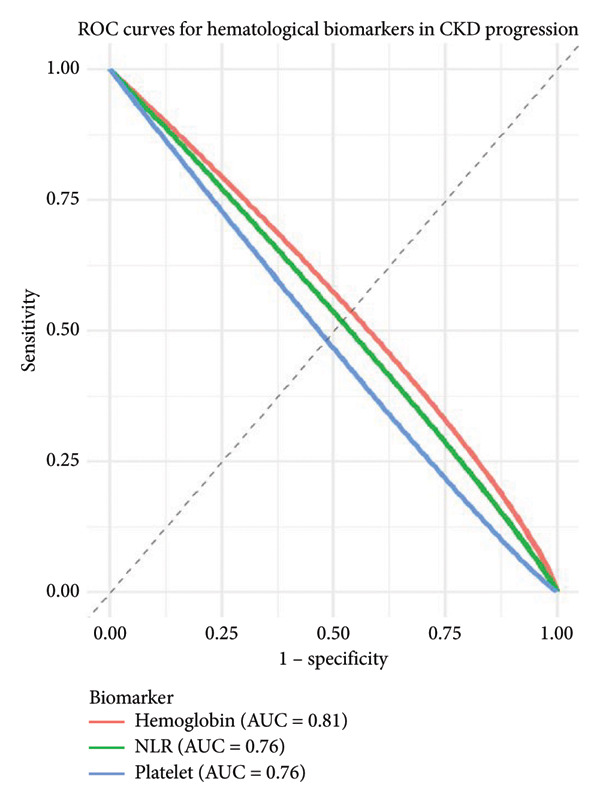
ROC curves predicting CKD progression.

Anemia (Hb < 12 g/dL for women and < 13 g/dL for men) was noted in 89 patients (74.2%). Its prevalence was found to rise from 33.3% in Stage 1 to 92.9% in Stage 5 (*p* < 0.001). Anemic patients demonstrated greater chances of developing LHV and fluid overload, per echocardiographic and radiographic notes present in the medical records of the patients. NLR values greater than 3.5 were recorded in 41 patients (34.2%) and were associated with higher CKD stage (*p* = 0.009) as well as with increased hospital admissions due to cardiovascular events (*p* = 0.015) as shown in Figure 2.

### 5.3. Predictive Value of Biomarkers

Multiple logistic regression analysis indicated Hb (OR: 0.72; 95% CI: 0.60–0.85; *p* < 0.001), NLR (OR: 1.43; 95% CI: 1.10–1.88; *p* = 0.006), and platelet counts (OR: 0.98; 95% CI: 0.96–0.99; *p* = 0.021) predicting progression to CKD Stage 5 independently from others. The ROC analysis revealed an AUC value for Hb of 0.81 (95% CI: 0.72–0.90), for NLR of 0.76 (95% CI: 0.66–0.85), and for platelet count of 0.68 (95% CI: 0.57–0.79) (see Table 3).

**Table 3:** Multiple logistic regression of hematological biomarkers predicting CKD progression.

Biomarker	Adjusted OR	95% CI	*p* value
Hemoglobin	0.72	0.60–0.85	< 0.001
NLR	1.43	1.10–1.88	0.006
Platelet count	0.98	0.96–0.99	0.021

These data suggested a moderate ability to predict especially by Hb and NLR as illustrated in Figure 2.

Figure 2 shows ROC curves for Hb, NLR, and platelet count predicting progression to CKD Stage 5. AUCs: Hb = 0.81 (95% CI 0.72–0.90), NLR = 0.76 (95% CI 0.66–0.85), and platelet count = 0.68 (95% CI 0.57–0.79).

The ROC analysis showed that Hb had an AUC of 0.81 (95% CI: 0.72–0.90), indicating good predictive power, NLR had an AUC of 0.76 (95% CI: 0.66–0.85), and platelet count had a moderate AUC of 0.68 (95% CI: 0.57–0.79).

### 5.4. Correlation Between Biomarkers and Kidney Function

The correlation between hematological biomarkers and kidney function, as assessed using eGFR, was investigated. A negative correlation between Hb concentration and eGFR (*r* = −0.70, *p* < 0.001) indicated that falling Hb concentration is associated with worsening kidney function. Similarly, a negative correlation of eGFR (*r* = −0.65, *p* < 0.001) was observed for RBC counts, indicating a decrease in RBCs with falling kidney function. Platelet count had a positive correlation with eGFR (*r* = 0.58, *p* < 0.01), indicating that higher platelet counts are associated with better kidney function. WBC count had some discrepancy, with a correlation of (*r* = 0.35, *p* = 0.014), which may be due to inflammation or infection compromising kidney function. Pearson correlation showed significant associations between biomarkers and eGFR, as illustrated in Table 4.

**Table 4:** Correlation of hematological biomarkers with kidney function (eGFR).

Biomarker	Correlation coefficient (*r*)	*p* value (*p*)	Interpretation
Hemoglobin	−0.70	< 0.001	Strong negative correlation
RBC count	−0.65	< 0.001	Moderate‐to‐strong negative correlation
Platelet count	**+0.58**	< 0.01	Moderate positive correlation
WBC count	+0.35	0.014	Weak positive correlation

*Note:* The bold values highlight biomarkers that show statistically significant and clinically meaningful correlations with kidney function, indicating relationships that are strong enough to be considered reliable.

Declining kidney function (lower eGFR) is significantly associated with reductions in Hb, RBCs, and platelets. The moderate inverse correlation with Hb supports its role as a predictive biomarker of disease severity. The positive correlation with WBC may reflect underlying inflammatory states in progressive CKD.

## 6. Discussion

In this study, the investigation focused on how some hematological biomarkers can predict the progression and complications of CKD populations. The results demonstrated significant associations between hematological parameters such as Hb, NLR, and platelet count with CKD progression. This implies that the routine assessment of these biomarkers might be useful in CKD management. Considering that anemia is largely associated with a decline in renal function, our study affirmed that Hb and RBC count levels decline significantly with increasing CKD stages, a finding that stood in agreement with other studies. Anemia occurs in CKD mainly because of defective erythropoietin production but can result from other causes such as inflammation or uremia [17]. The prevalence of anemia increased from 33.3% in Stage 1 to 92.9% in Stage 5, with Hb levels strongly correlated with poorer kidney functions, in concordance with other studies [18]. Besides, multiple logistic regression has shown that Hb independently predicts progression to CKD Stage 5, therefore further alluding to its prognostic significance in CKD management.

Raised NLR, as a marker for systemic inflammation, had a significant relationship with higher CKD stages and cardiovascular complications, including LVH and fluid overload. Inflammation pathways have gained enhanced recognition as critical contributors to CKD progression and cardiovascular morbidity. The association of NLR with CKD complications supports its role as an inflammatory biomarker, and previous studies have shown that a higher NLR is indicative of poor outcomes in patients with CKD [19]. Platelet count also correlated significantly with kidney function, with thrombocytopenia being observed more frequently in advanced stages of CKD. The relationship between platelets and CKD progression is multifactorial and might be influenced by factors such as uremia, inflammation, and also the use of anticoagulant therapies in advanced stages [20]. While the pathophysiological mechanisms are yet to be elucidated, the results of our study align with those demonstrated in the literature that link platelet abnormalities with renal dysfunction [21, 22]. This study highlights routine hematological parameters as potentially useful for aiding in the prognosis of CKD. The Hb concentration of CKD patients, NLR, and platelet count are cheap tests that could easily be incorporated into the routine CKD management protocols for risk stratification and therapeutic intervention [23, 24]. For instance, early detection of anemia and inflammation can initiate therapeutic interventions such as erythropoiesis‐stimulating agents or anti‐inflammatory agents, which may slow down the process of CKD [25, 26].

The outcomes of this study have found resonance with global research that has emphasized hematological markers’ relevance in CKD risk stratification and prognostication. For instance, studies in developed and developing countries have demonstrated that a decrease in Hb level may mirror renal anemia or stand as an independent predictor of cardiovascular adverse events and mortality in CKD patients [27, 28]. The same goes for the predictive value of NLR in different cohorts, including large‐scale meta‐analyses establishing an association between high NLR and rates of hospitalization and death among patients with advanced kidney disease [29, 30]. Platelet derangements of whatever form are being increasingly studied on how these worsen uremia‐induced dysfunction and thromboinflammatory states that would otherwise hamper CKD‐related complications [31, 32]. These arguments strengthen the current findings and also indicate that even in a resource‐poor environment, simple blood‐based markers can go a long way to be of clinical use. Intervening on these markers could go a long way in delaying disease progression, thus reducing the burden of dialysis and transplantation. Future studies could look into integrating hematological markers with renal function indices, inflammatory cytokines, and emerging genetic biomarkers to provide strong prediction models that would be both cheap and scalable in a low‐income setting.

### 6.1. Study Limitations

This study was limited by its retrospective design, which is contingent on the accuracy and adequacy of existing medical records. While a wide spectrum of CKD stages was included, this study was conducted at a single center, and the results may not be generalizable to other settings or populations. Other future multicenter trials are needed to validate findings and examine the long‐term effects of hematological biomarkers on CKD complications and progression. In addition, even though this study was on widely available biomarkers, the future would hold potential for study on how these are to be coupled with other molecular and genetic biomarkers in order to enhance predictive models. This would allow for more tailored management of CKD, particularly in low‐resource settings where advanced diagnostic tools are not available.

## 7. Conclusion

Hematological biomarkers such as Hb and NLR are good indicators of CKD complications and progression among adult patients. Their use in routine clinical practice could assist in enhancing early diagnosis of worsening renal function and improve more successful CKD management, especially for resource‐limited settings.

## Disclosure

All authors have given their consent for publication of this article. This research has been published as a preprint on Research Square_https://www.researchsquare.com/article/rs-5728649/v1.

## Conflicts of Interest

The authors declare no conflicts of interest.

## Author Contributions

All authors reviewed this article.

## Funding

No funding was received for this research.

## Data Availability

The data of the findings of this study are all shared within this article.
